# Comparative electrostatic mapping of ATP synthase reveals species‐dependent axial asymmetry

**DOI:** 10.1002/pro.70843

**Published:** 2026-11-14

**Authors:** Islam K. Matar, Peyman Fahimi, Jean‐Nicolas Vigneau, Chérif F. Matta

**Affiliations:** ^1^ Department of Chemistry & Physics Mount Saint Vincent University Halifax Nova Scotia Canada; ^2^ Department of Chemistry Saint Mary's University Halifax Nova Scotia Canada; ^3^ Department of Chemistry Université Laval Québec Québec Canada; ^4^ Department of Mathematics and Statistics Dalhousie University Halifax Nova Scotia Canada

**Keywords:** ATP synthase, comparative protein electrostatics, cross‐species comparison, membrane dielectric effects, mycobacterial ATP synthase, Poisson–Boltzmann equation, protein axial electrostatic asymmetry, proton motive force (pmf)

## Abstract

ATP synthase operates within a highly structured electrostatic environment, but comparative information on its intrinsic protein electrostatics across species remains limited. To investigate species‐dependent variations in ATP synthase electrostatics, 178 experimentally determined structures from 17 species are analyzed using a consistent Poisson–Boltzmann workflow. The calculations reveal reproducible species‐dependent axial electrostatic asymmetries across the F_o_–F_1_ complex, with axial electrostatic potential differences of approximately 10–20 mV between the proton‐entry and proton‐exit sides of the F_o_ sector in several taxa. These differences are calculated by subtracting the axial‐window average of the plane‐averaged electrostatic potential over the entry‐side window (*z* = −10 to +10 Å) from the corresponding average over the exit‐side window (*z* = +50 to +70 Å). The *z*‐coordinate specifies the position of a plane perpendicular to the rotational axis of the c‐ring, which coincides with the long axis of ATP synthase; *z* = 0 defines the plane passing through the proton‐entry site, with positive *z* directed toward the proton‐exit side. The five available *Homo sapiens* structures display a consistent axial electrostatic orientation, whereas broader variation is observed across the full multi‐species dataset; these two observations reflect within‐species and across‐species comparisons, respectively. Membrane‐mimicking low‐dielectric slabs amplify the profiles and demonstrate the sensitivity of the electrostatic landscape to dielectric environment. An opposing axial electrostatic orientation observed in human ATP synthase raises the possibility of a slight reduction in the fraction of proton‐motive‐force energy conserved as ATP, although this hypothesis remains to be tested experimentally. We interpret these quantities as static, structure‐derived electrostatic descriptors, rather than demonstrated additive contributions to the proton‐motive force. The results provide a comparative electrostatic atlas of ATP synthase and suggest testable hypotheses concerning local proton‐pathway energetics, structural evolution, and inhibitor sensitivity.

## 1 INTRODUCTION

ATP synthase is the rotary enzyme that catalyzes the synthesis of ATP using the proton motive force (pmf) across energy‐transducing membranes. The core concept of Mitchell's chemiosmotic theory (Mitchell, 1961; Mitchell, 2011) is that the free energy from oxidative metabolism or photosynthetic electron transport, stored as an electrochemical proton gradient across the inner mitochondrial membrane (IMM), bacterial plasma membrane, or chloroplast thylakoid membrane, is used by the enzyme to drive ATP formation.

During mitochondrial aerobic respiration, electron transport through complexes I–IV of the electron transport chain (ETC) drives protons (H^+^) from the mitochondrial matrix to the intermembrane space (IMS), creating and maintaining an electrochemical gradient across the inner membrane. The resulting proton motive force has two components: an electrical potential difference (voltage), ΔΨ (matrix negative, IMS positive), and a chemical gradient (in proton concentration, resulting in a pH difference across the membrane, ΔpH = pH_in_ – pH_out_). The pmf (in volts) is defined (Rosenberg, 1987):

(1) $$\mathrm{pmf}=\Delta \Psi -\frac{2.303\;\mathrm{RT}}{F}\;\mathrm{\Delta pH}$$

where R is the gas constant (8.314 $J.{\mathrm{mol}}^{-1}.K^{-1}$), T is the absolute temperature, and F is Faraday's constant (96,485 $C.{\mathrm{mol}}^{-1}$), and $\frac{2.303\;\mathrm{RT}}{F}\approx 0.05916\;V$ (i.e., ≈59 mV per pH unit; Rosenberg, 1987) at *T* = 298 K (and ≈62 mV at *T* = 310 K) for a ΔpH of 1. By convention, ΔΨ is defined as the electrical potential in the interior (e.g., mitochondrial matrix) minus the potential outside (e.g., mitochondrial intermembrane space). This ΔΨ is the electrical component of the transmembrane electrochemical potential (not the full pmf). That is, ΔΨ is the net electrical potential difference generated by charge separation across the membrane. In the chemiosmotic expression it is combined with the ΔpH term to define the pmf. Reported mitochondrial ΔΨ ranges from approximately −90 to −225 mV (negative inside) (Liesa & Shirihai, 2013) which is postulated to be actively regulated through an electrical feedback mechanism (Fahimi et al., 2025). For chloroplasts, ΔΨ values as low as −30 mV have been reported, while in bacteria ΔΨ typically ranges from −90 to −180 mV (Cheuk & Meier, 2021). The relative contributions of ΔΨ and ΔpH to the total pmf vary widely across species and physiological states (Cheuk & Meier, 2021).

The free energy change for a mole of protons moving down the electrochemical gradient (from the IMS back into the mitochondrial matrix) is given by:

(2) $$\Delta G_{H^{+}}=F\;\Delta \Psi -2.303\;\mathrm{RT}\;\mathrm{\Delta pH},$$

whereby, ΔΨ = Ψ_in_ – Ψ_out_. This formula gives the signed Gibbs energy change accompanying a proton moving inward, that is, Δ*G* < 0 indicates a spontaneous flow of protons. Under typical mitochondrial conditions (ΔΨ ≈ −150 to −200 mV and ΔpH ≈0.5–1), the proton motive force corresponds to ~20–30 kJ·mol^−1^ of proton (Battogtokh et al., 2018; Rosenberg, 1987). Throughout this paper, “*in*" or "*exit*” and “*out*” or "*entry*" designate the proton‐exit and proton‐entry sides, respectively, during ATP synthesis: the matrix and IMS in mitochondria, the cytosol and extracellular medium in bacteria, and the stroma and thylakoid lumen in chloroplasts.

Although Δ*G*°′ for ATP synthesis (${\mathrm{ADP}}^{3-}+P_{i}^{2-}+H^{+}⟶{\mathrm{ATP}}^{4-}+H_{2}O$) is +30.5 kJ·mol^−1^ under standard biochemical conditions, the high intracellular [ATP]/[ADP][P_i_] ratio shifts the effective free energy of ATP formation to approximately 50–60 kJ·mol^−1^ under physiological conditions (Macdonald & Ashby, 2025; Seyfried et al., 2020; Veech et al., 1979), which will be referred to as Δ*G*_ATP_. The proton motive force must therefore supply at least this amount of free energy per ATP synthesized, in addition to unavoidable dissipative losses.

In mammalian mitochondria, rotor stoichiometry and metabolite transport together yield an effective requirement of approximately 3.5–4 protons per ATP synthesized and exported to the cytosol (Garrett & Grisham, 2013). This value reflects both c‐ring geometry and the electrogenic cost of ATP/ADP exchange and phosphate import.

ATP synthase is a proton‐driven rotary nanomachine in which translocation through the membrane‐embedded F_o_ sector generates a torque on the c‐ring and central γ‐shaft, thereby powering ATP synthesis in F_1_ (Boyer, 1999). At a typical mitochondrial proton motive force (of magnitude ≈200 mV), each proton delivers ~0.2 eV (Matar et al., 2025), whereas the intact enzyme rotates at ~100–150 revolutions per second (rps) under physiological conditions (and up to ~650 rps in thermophilic systems (Ueno et al., 2005)). In contrast, treating the F_o_ c‐ring as a quantum mechanical rigid rotor predicts minimum angular velocities (for the first non‐zero quantum number [*l* = 1]) on the order of 10^4^–10^5^ rps, one to three orders of magnitude above biological operation (Matar et al., 2025). This is consistent with the experiments of Lill et al., whose data imply a rotation rate of ~4 × 10^4^ rps for isolated F_o_ from chloroplast ATP synthase (Lill et al., 1987).

Lill et al. report that the “*average*” proton conductance across all isolated F_o_ channels in their experiments is approximately 6200 H^+^.s^−1^, a value that includes both active and inactive ATP synthases. However, when considering only the active F_o_ channels (effectively a single‐molecule–level analysis based on statistical evaluation), they obtain a conductance of ~6 × 10^5^ H^+^.s^−1^ per active isolated F_o_ channel, equivalent to ~4 × 10^4^ rps falling exactly within the range of quantum mechanical calculations (Lill et al., 1987; Matar et al., 2025).

Energetically, ATP synthase operates in a strongly overdamped regime in which ~75–83% of proton free energy is captured in ATP and ~ 17–25% is dissipated, predominantly through internal mechanical friction (Matar et al., 2025). In this context, even modest intrinsic electrostatic potential differences of 10–20 mV could be relevant to this energy partitioning, provided that they are sampled locally by protons during translocation through the F_o_ sector. Such potentials are protein‐generated electrostatic features that may, in principle, bias local protonation, proton release, proton transfer, or coupling efficiency by approximately 1–2 kJ·mol^−1^ per proton.

Vigneau et al. (Vigneau et al., 2022) proposed that ATP synthase carries an intrinsic molecular electrostatic potential (ESP) with a dipolar orientation perpendicular to the membrane. In all five structures examined in that study, the axial ESP is found to favour proton translocation (Vigneau et al., 2022). The present work extends that analysis to 178 ATP synthase structures from 17 species and shows that the sign and magnitude of this axial electrostatic asymmetry are species‐dependent. As will be shown in this study, the available human mitochondrial ATP synthase structures exhibit an axial ESP orientation opposite to that observed in the previously studied enzymes.

## 2 ATP SYNTHASE'S INTRINSIC ELECTROSTATIC POTENTIAL (ESP)

Vigneau et al. (Vigneau et al., 2022) show that F_o_‐F_1_ ATP synthase possesses a protein‐intrinsic axial electrostatic potential that could, in principle, influence the local electrostatic environment encountered by translocating protons. Figure 1 depicts our hypothesis that the enzyme's electric field's component perpendicular to the membrane plane and its associated electrostatic potential may be parallel (constructive, Figure 1 (left)) or antiparallel (destructive, Figure 1 (right)) to the electrical part of the pmf.

**FIGURE 1:**
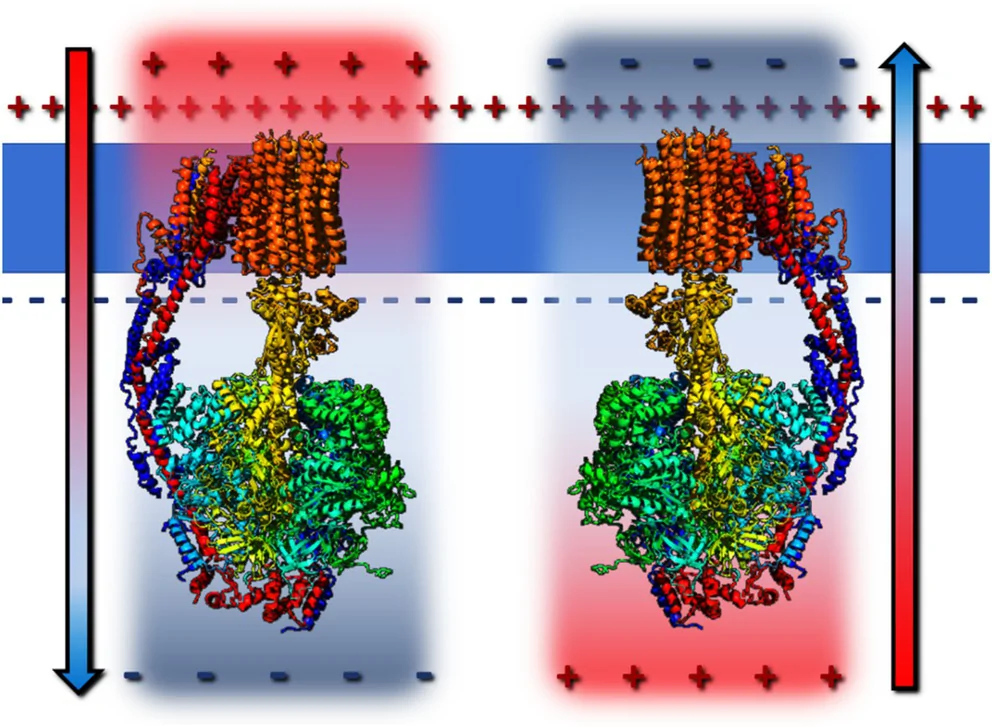
Schematic illustration of the hypothesis: Two opposite axial electrostatic polarity (orientation) classes in F_o_‐F_1_ ATP synthase: Positive‐entry axial orientation (*left*) (constructive) and negative‐entry axial orientation (*right*) (destructive). The membrane bilayer (blue slab) contains the F_o_ sector.

Vigneau et al. (Vigneau et al., 2022) examined five structures from *Paracoccus denitrificans*, *Bacillus* sp. (bacteria), *S. cerevisiae* (yeast), *Y. lipolytica* (fungus), and *Sus scrofa* (mammal). This analysis is extended to 178 structures retrieved from the Protein Data Bank (RCSB Protein Data Bank, 2025). Each structure was prepared with PDB2PQR, which was used to assign AMBER force‐field atomic partial charges and radii and to generate PQR files (Salomon‐Ferrer et al., 2013). Electrostatic potentials were then computed by numerically solving the finite‐difference linearized Poisson–Boltzmann equation (LPB) using the Adaptive Poisson–Boltzmann Solver (APBS) (Jurrus et al., 2018) with pH = 7.0, T = 298 K (25°C), and with the dielectric constant values: ε_solvent_ = 78.5, ε_protein interior_ = 6, and where applicable, ε_membrane_ = 4. In all calculations, a bulk ionic strength of 150 mM NaCl was maintained (Baker et al., 2001). Calculated electrostatic potential grids surrounding each structure were analyzed using an in‐house Nextflow/Python pipeline (see Methodology). The linearized formulation of the PB equation is formally valid in the regime $|e\Psi |\ll k_{B}T$ (when ∣Ψ∣ ≪ ∼25.7 mV at the calculation temperature) since the nonlinear ion‐density term $\sinh\left(e\Psi /k_{B}T\right)$ may be approximated to first order (Baker et al., 2001; Holst et al., 2000; Honig & Nicholls, 1995). Under physiological screening conditions (Debye length ≈8 Å at 150 mM, *vide infra*), nonlinear corrections are reduced by ionic screening, and LPB provides an accurate first‐order description of biomolecular electrostatics for comparative analyses. Importantly, the linearized equation yields a linear elliptic partial differential equation that can be solved via a single sparse linear system, avoiding the iterative nonlinear solution procedures required by the full Poisson–Boltzmann equation. This substantially improves the numerical robustness and reduces computational costs. Because all structures were treated under identical dielectric and ionic conditions (150 mM NaCl), the LPB calculations provide a consistent basis for comparison, though neglected nonlinear effects may vary among structures. Importantly, electrostatic potential *differences* may be less sensitive to systematic computational errors than *absolute* potentials. Writing Ψ_calc_(**r**) = Ψ_ref_(**r**) + δ(**r**), where δ(**r**) represents the modeling error, the corresponding error in the axial potential difference is ΔΨ_calc_ − ΔΨ_ref_ = ⟨δ⟩_exit_ − ⟨δ⟩_entry_, where the brackets denote the successive plane and axial‐window averaging procedures used in this study. Thus, errors that are similar in both windows may partially cancel, improving the robustness of the calculated differences. Nevertheless, total cancellation is not guaranteed and does not completely eliminate the limitations of the LPB approximation.

All five structures previously studied (Vigneau et al., 2022) exhibit constructive contributions from the ESP of ATP synthase (${\Delta \Psi }_{\mathrm{ATP}\;\text{synthase}}$) to the pmf. For example, the plane‐averaged electrostatic potential $\left(\left\langle \Psi_{\mathrm{ATP}\;\text{synthase}}\left(x,y\;|\;z\right)\right\rangle \right)$ of *Yarrowia lipolytica* ATP synthase (PDB 5FL7) exhibits a net decrease along the *z*‐axis, from the intermembrane space toward the mitochondrial matrix. The potential is positive near the upper vestibule and becomes progressively more negative along the proton pathway toward the matrix (Figure 2). The *z*‐axis coincides with the c‐ring rotational axis, with *z* = 0 defining the plane containing the proton‐entry site perpendicular to this axis, and the positive *z‐*direction is chosen to point toward the matrix (proton exit). The average ESP of this fungus is positive near the upper vestibule and becomes progressively more negative along the proton pathway toward the matrix. The (average) ATP synthase‐associated axial voltage between the point of entry and exit of the proton into and from the membrane domain, that is, the enzyme‐dependent voltage, is defined:

(3) $$\Delta \left\langle \Psi_{\mathrm{ATP}\;\text{synthase}}\right\rangle \equiv \left\langle \Psi_{\mathrm{ATP}\;\text{synthase}}\left(x,y|z_{\text{exit}}\right)\right\rangle \;-\left\langle \Psi_{\mathrm{ATP}\;\text{synthase}}\left(x,y|z_{\text{entry}}\right)\right\rangle ,$$

where ${\Delta \Psi }_{\mathrm{ATP}\;\text{synthase}}$ is defined analogously to the chemiosmotic membrane potential as Ψ_exit_ − Ψ_entry_ (given that $\Psi_{\mathrm{exit}}<\Psi_{\mathrm{entry}}$ under normal oxidative phosphorylation). The angular brackets indicate averaging over an *xy*‐plane (see Methods), and where *z*_entry_ and *z*_exit_ are the approximate *z*‐coordinates on the entry/out side and on the exit/in side, respectively. For clarity, $\Delta \left\langle \Psi_{\mathrm{ATP}\;\text{synthase}}\right\rangle$ means the difference of the averages and not the average of the differences. For notational simplicity, henceforth angular brackets denoting spatial averaging are omitted from the enzyme‐linked potential differences throughout the text, except where needed to avoid ambiguity.

**FIGURE 2:**
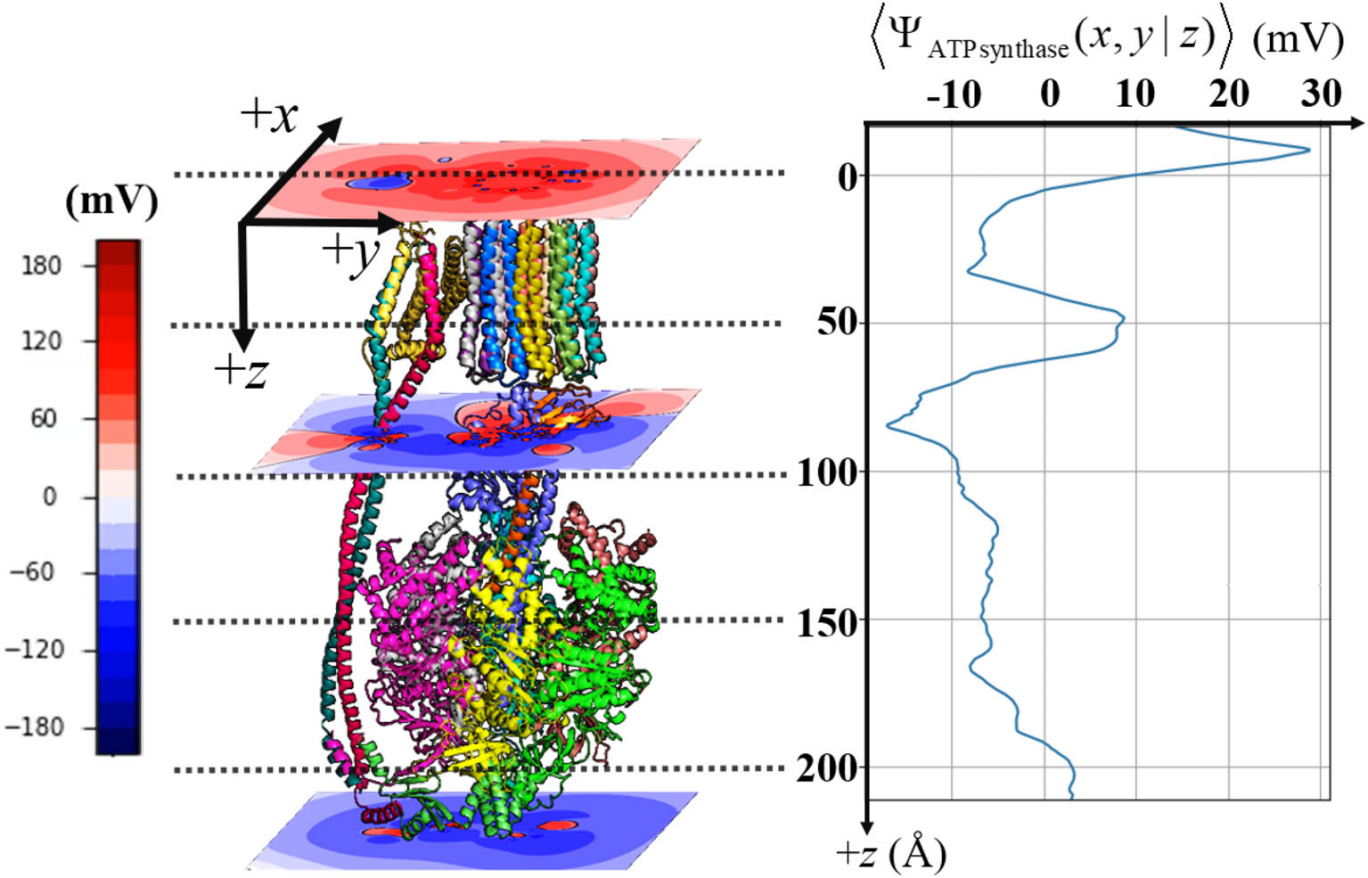
Average electrostatic potential of ATP synthase of *Yarrowia lipolytica* (PDB Code # 5FL7) per cross‐sectional *xy*‐planes as a function of the *z*‐coordinate (the long axis of the protein perpendicular to the inner mitochondrial membrane). *Left*: The enzyme is shown with three representative *xy*‐slices colored by electrostatic potential (red, positive; blue, negative) with the color‐scale showing the values of the electrostatic potential in mV. A Cartesian coordinate system is indicated with +*z* increasing downward. For clarity, the *z*‐axis is shown displaced to the left from its actual position along the central rotational axis of the ATP synthase c‐ring. Dotted lines mark the approximate *z*‐positions of the *xy*‐planes perpendicular to *z*. *Right*: Profile of the average potential per *xy*‐plane, $\langle \Psi_{\mathrm{ATP}\;\text{synthase}}\left(x,y\;|\;z\right)\rangle$ in mV plotted against *z* in Å using the same +*z*‐down convention. This curve summarizes how the electrostatic potential varies along the axis perpendicular to the membrane.

The proton translocation “reaction” path is written as:

(4) $$H_{\mathrm{entry}}^{+}⟶H_{\mathrm{exit}}^{+}.$$

In all five previously studied organisms (Vigneau et al., 2022), ${\Delta \Psi }_{\mathrm{ATP}\;\text{synthase}}$ has the same sign as the transmembrane potential (${\Delta \Psi }_{\text{membrane}}$) reinforcing the electric component of the pmf.

The enzyme‐linked electrostatic potentials reported by Vigneau et al. correspond to energy shifts of approximately 1–3 kJ·mol^−1^ of protons. Given that cellular ATP synthesis requires ~50–60 kJ·mol^−1^ per ATP and ~3–4 protons per ATP in mammalian mitochondria (depending on c‐ring stoichiometry and transport costs), the energetic effect can be non‐negligible. Because Δ*G*_ATP_, proton stoichiometry, and ATP yield per substrate molecule vary with organism, metabolic state, and carbon source (Brand, 2005; Turina et al., 2016), the resulting energetic correction is system‐dependent. Under typical mitochondrial conditions, a hypothetical electrostatic energy shift of 1–3 kJ·mol⁻^1^ of protons would represent approximately 3–15% of the proton‐motive‐force energy (20–30 kJ·mol⁻^1^ per proton), if experienced during translocation.

The recent determinations of the structure of the human ATP synthase (Lai et al., 2023; Zhang et al., 2024) prompted us to extend the analysis to a broader range of species. Hence, 178 electron microscopy and X‐ray crystallographic structures of ATP synthase from 17 different organisms (including *Homo sapiens*) were obtained from the Protein Data Bank (RCSB Protein Data Bank, 2025), including the five previously studied structures. This dataset includes structures of 17 species which, by kingdom, include *Animalia* (mitochondrial) [*Bos taurus* (12 structures), *Homo sapiens* (5), *Ovis aries* (1), and *Sus scrofa* (2)]; *Bacteria* (plasma membrane bound) [*Acinetobacter baumannii* (3 structures), *Bacillus* sp. (3), *Escherichia coli* (20), *Mycobacterium tuberculosis* (3), *Mycolicibacterium smegmatis* (24), and *Paracoccus denitrificans* (1)]; *Fungi* (mitochondrial) [*Ogataea angusta* (3 structures), *Saccharomyces cerevisiae* (39), and *Yarrowia lipolytica* (1)]; Plantae (chloroplast membrane bound) [*Spinacia oleracea* (12)], green algae (mitochondrial) [*Polytomella* sp. (45 structures)]; and *Protista* [*Euglena gracilis* (mitochondrial) (3 structures) and *Toxoplasma gondii* (mitochondrial) (1)]. These groupings do not, by themselves, establish a relationship between ATP synthase electrostatics and phylogeny or physiology. They suggest, however, that ATP synthase electrostatics may vary with membrane type, c‐ring composition, lipid environment, respiratory strategy, and pmf partitioning. The species‐dependent classes identified here are to be taken as *comparative electrostatic phenotypes* rather than as functional categories. Whether similar electrostatic descriptors cluster along specific evolutionary branches, membrane types, or metabolic characteristics requires a larger phylogenetically controlled dataset and experimental measurements of pmf, proton flux, and ATP synthesis.

All 178 structures were subjected to the same protocol (see “Methods”). The ${\Delta \Psi }_{\mathrm{ATP}\;\text{synthase}}$ values obtained in full aqueous solvation provide a reference for assessing the effects of membrane dielectric constant, which can alter the magnitudes and ordering of the calculated electrostatic descriptors. Plots of the enzyme‐dependent plane‐averaged voltage as a function of the *z*‐coordinate ($\left\langle \Psi_{\mathrm{ATP}\;\text{synthase}}\right\rangle$) are shown in Figure 3, while Figure 4 compares the mean axial electrostatic potential differences (± standard deviation (SD)) across 17 species, calculated with and without a membrane‐mimicking dielectric slab.

**FIGURE 3:**
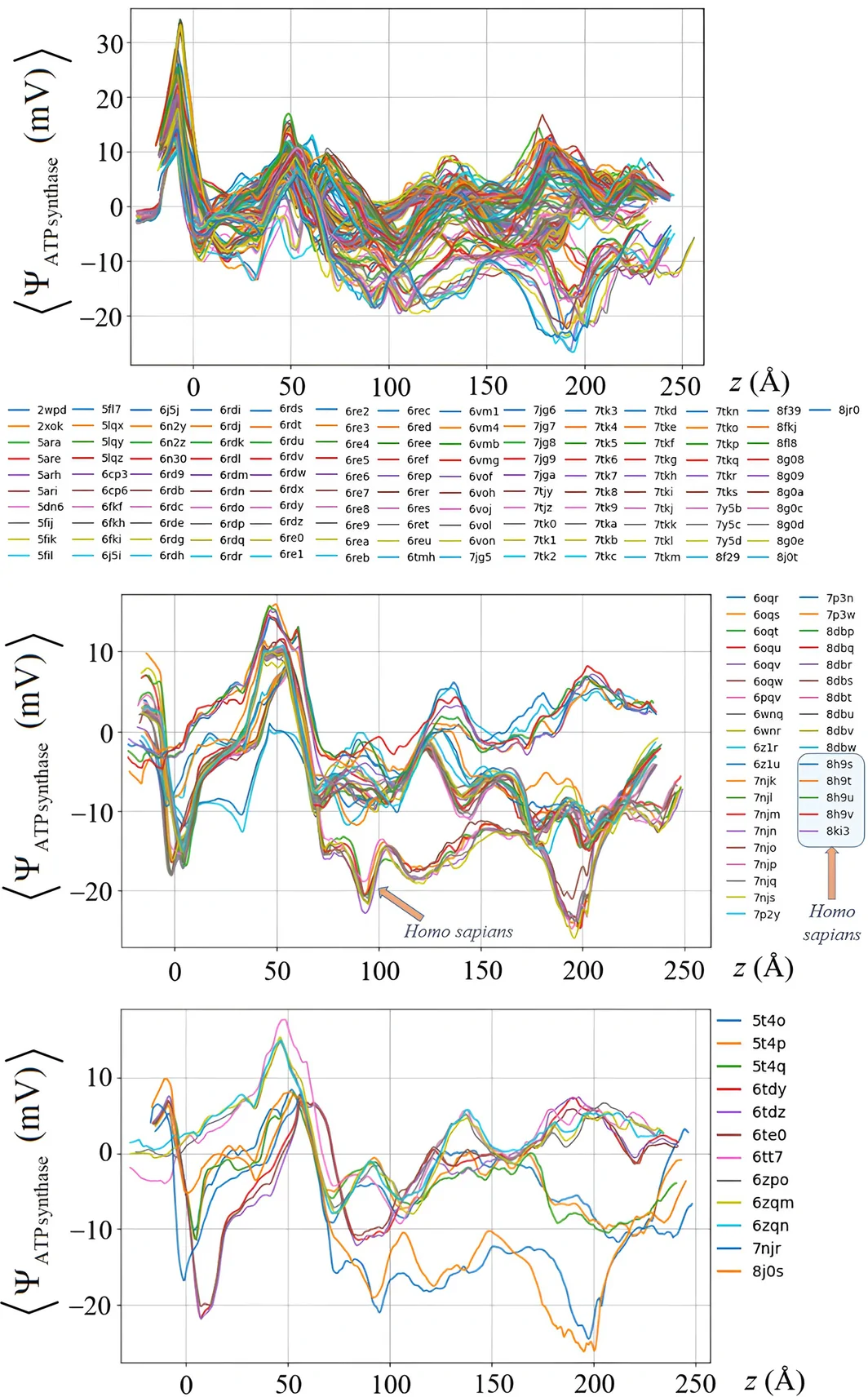
Axial plane‐averaged electrostatic potentials for the 178 ATP synthase structures (PDB codes in legend). Each trace shows ⟨Ψ(*x*,*y*|*z*)⟩ (mV), obtained by averaging the electrostatic potential over planes perpendicular to the molecular long (*z*‐) axis. Profiles are grouped into three panels according to their low‐*z* (entry/out‐side) behavior: (top) initial positive band; (middle) pronounced negative trough at entry; (bottom) ambiguous: near‐zero to moderately negative or positive entry. All calculations were performed in bulk aqueous medium (ε = 78.5) with 150 mM NaCl and no explicit membrane. The aqueous profiles provide a reference for comparison with the membrane‐embedded calculations. The five *Homo sapiens* structures (indicated in the legend) cluster within the negative‐entry group and all five exhibit the same sign and orientation of the axial potential gradient at the entry/out side (IMS interface), i.e., a negative potential upon entry from low *z*, followed by recovery toward positive values on the mitochondrial matrix side. Only ten distinct line colors are used in the main figure; individual traces for all 178 structures are provided in the Data S1.

**FIGURE 4:**
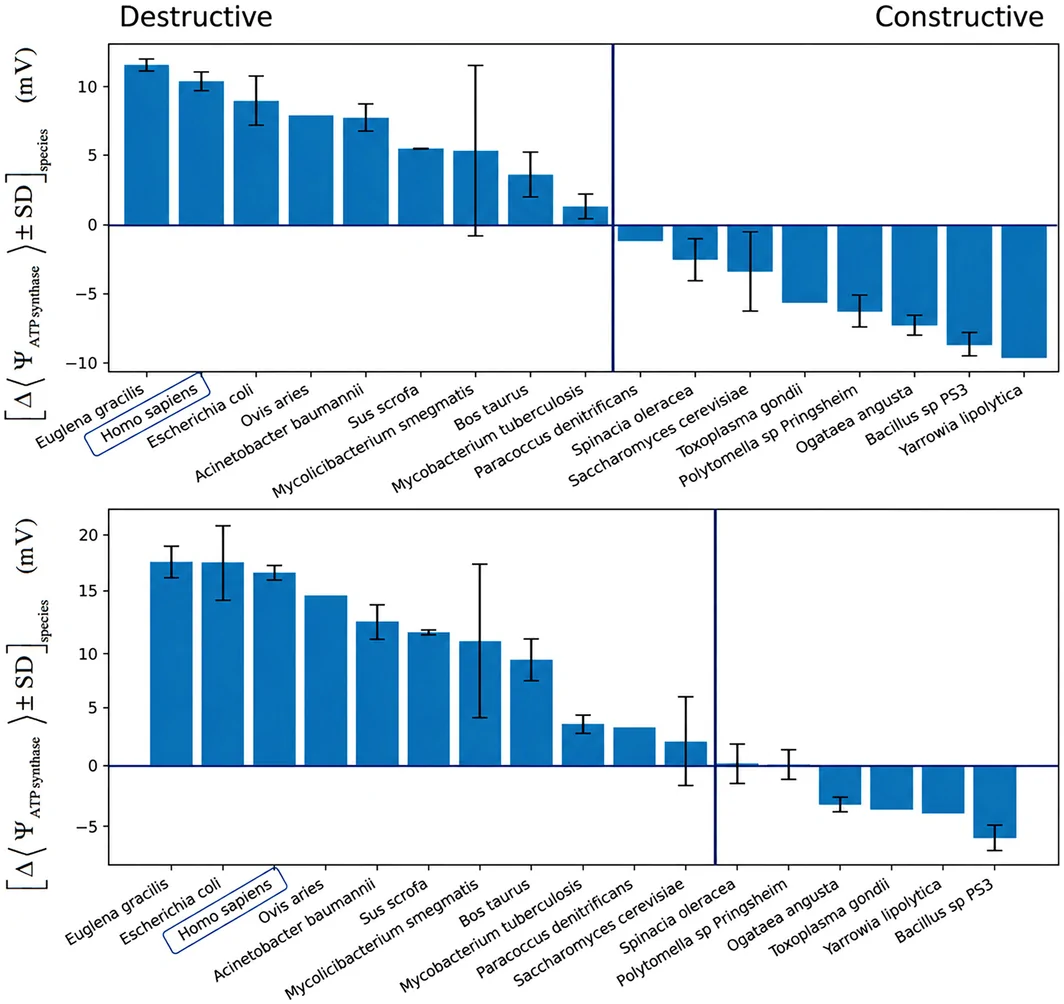
Species‐dependent enzyme‐linked voltage in aqueous and membrane‐embedded models. Top: ATP synthase–dependent potential difference (${\Delta \Psi }_{\mathrm{ATP}\;\text{synthase}}$) computed in full aqueous medium (150 mM NaCl) for 17 species calculated as the difference between the mean potentials at the proton exit‐side (*z* = +50 to +70 Å) and at the entry‐side (*z* = −10 to +10 Å) windows. Bottom: Corresponding ${\Delta \Psi }_{\mathrm{ATP}\;\text{synthase}}$ values after embedding the F_o_ sector in a planar dielectric slab (ε = 4) to mimic the membrane core. The slab extends from *z* = +10 to +50 Å. In both bar‐graphs, “destructive” (negative potential at proton entry) and “constructive” (positive potential at proton entry) are defined according to their opposition or alignment to the physiological membrane‐potential sign, respectively (Equation 3). Bars represent species means, with error bars indicating ±1 standard deviation (SD) across structures where multiple structures are available. Comparison of the two bar graphs isolates the influence of membrane dielectric effects on the enzyme‐linked voltage (see also Figure 5).

Figure 3 overlays the 178 plane‐averaged electrostatic potential profiles $\left\langle \Psi_{\mathrm{ATP}\;\text{synthase}}\right\rangle$ along the enzyme's long *z*‐axis (Figures 1, 2). Structures are classified according to the plane‐averaged electrostatic‐potential profile in a membrane‐proximal entry‐side window (*z* = −20 to +20 Å). The classification is based on the first prominent peak or trough in the plane‐averaged ESP profile within this window. When no clear peak or trough was detected, the mean plane‐averaged potential over the same window was used (see Supporting Information (SI)). This window was chosen to represent the electrostatic environment encountered by a proton as it approaches the F_o_ sector during ATP synthesis. On this basis, three groups emerge: positive‐entry profiles (⟨Ψ⟩ > +10 mV; 131 structures), ambiguous‐to‐moderate profiles (0 to +10 mV; 12 structures), and negative‐entry profiles (⟨Ψ⟩ < 0 mV; 35 structures, including all five human structures). The classification into three categories in Figure 3 was therefore based on this finite averaging window. A small number of borderline cases lie near the threshold between categories; minor smoothing or structural differences may therefore make some curves appear visually closer to an adjacent panel without altering the sign‐based grouping criterion. This classification is therefore descriptor‐based. The groups are defined by the sign and magnitude of the plane‐averaged entry‐side electrostatic potential in a fixed axial window, applied uniformly to all structures. This differs from recent machine‐learning and multidimensional embedding approaches to protein electrostatic classification, which compare higher‐dimensional electrostatic features across protein sets (Wilson et al., 2024). Our aim here is narrower being limited to define an ATP‐synthase‐specific axial electrostatic descriptor along the membrane‐normal direction.

The axial electrostatic asymmetry descriptor ${\Delta \Psi }_{\mathrm{ATP}\;\text{synthase}}$ was calculated by averaging the ESP over a 20 Å window on each side of the membrane to compare species based on the potential difference. The ESP was averaged from *z* = −10 to +10 Å on the entry‐side window and from *z* = +50 to +70 Å on the exit‐side window, and ${\Delta \Psi }_{\mathrm{ATP}\;\text{synthase}}$ is defined as the difference between these two averages. The same procedure was applied to each structure and the resulting values were averaged across all structures available for each species. Figure 4 presents the species means, with error bars indicating ± SD.

In fully aqueous medium (ε = 78.5, 150 mM NaCl; Figure 4 (top)), ${\Delta \Psi }_{\mathrm{ATP}\;\text{synthase}}$ is typically modest (within ±10 mV), indicating that the intrinsic protein electrostatic potential alone generates a small axial voltage. In our previous work (Vigneau et al., 2022) we calculated the potential difference between two points at the beginning and end of the c‐ring, whereas here we average over 20 Å windows at both sides of the membrane, which explains the small numerical discrepancies between the results of the two studies. Embedding the F_o_ sector in a membrane‐mimicking dielectric slab (ε = 4, spanning *z* = +10 to +50 Å; Figure 4 [bottom]) alters the magnitude of ${\Delta \Psi }_{\mathrm{ATP}\;\text{synthase}}$ (spanning approximately 21 mV without the membrane to 24 mV with the membrane representing slab) and, in borderline cases, alters its species‐dependent axial electrostatic orientation. This comparison isolates the dielectric focusing effect of the membrane, that is, compression of electric field lines within the low‐dielectric slab at the membrane–water interfaces.

Although the insertion of a low‐dielectric slab (ε = 4) generally amplifies the axial field through dielectric focusing, it does not act as a uniform multiplicative rescaling of the enzyme‐linked voltage as 150 mM NaCl is included. This nonuniform response arises from the interplay between dielectric boundaries and ionic screening, as described by the linearized Poisson–Boltzmann equation:

(5) $$\nabla \cdot \left[\varepsilon_{r}\left(r\right)\nabla \Psi \left(r\right)\right]-\varepsilon_{r}\left(r\right)\kappa^{2}\left(r\right)\Psi \left(r\right)=-\frac{\rho_{\text{fixed}}\left(r\right)}{\varepsilon_{0}},$$

where *ρ*_fixed_ is the fixed charge density (C.m⁻^3^), defined as

(6) $$\rho_{\text{fixed}}\left(r\right)\equiv \sum_{i=1}^{N_{m}}\;q_{i}\;\delta^{\left(3\right)}\left(r-r_{i}\right),$$

where *N_m_* is the number of atomic point charges, *q_i_* is the partial charge of atom *i* (in coulombs), $\delta^{\left(3\right)}$ is a three‐dimensional Dirac delta function (with units of m^−3^), $\varepsilon_{r}\left(r\right)$ is the relative dielectric constant, *κ* is the inverse Debye screening length (m⁻^1^), and where the screening term *κ* ^2^Ψ operates only within ion‐accessible solvent regions (*κ* = 0 inside the membrane slab since there are no mobile ions) and depends on the Debye length associated with the salt concentration. Because the solution remains sensitive to the detailed spatial arrangement of charges and dielectric discontinuities, the change from fully aqueous to slab‐embedded conditions can perturb the window‐averaged axial potentials in a structure‐dependent manner. Consequently, rank ordering of ${\Delta \Psi }_{\mathrm{ATP}\;\text{synthase}}$ across species need not be preserved, even though the underlying equation is linear. In contrast, in vacuum or in salt‐free water, one would expect the slab primarily to produce a more uniform dielectric focusing effect with greater preservation of ordering (Baker et al., 2001; Honig et al., 1986; Honig & Nicholls, 1995; Im et al., 1998; Sharp & Honig, 1990).

Electrolyte screening may moderate the increase in ΔΨ upon membrane embedding. In salt‐free water (*κ* = 0), the low‐dielectric slab would primarily act through dielectric focusing, potentially increasing the axial potential difference without competing screening effects. At 150 mM NaCl, however, the linearized Poisson–Boltzmann term introduces Debye screening with a characteristic decay length of ~8 Å. (At 298 K, the Debye screening length for a 1:1 electrolyte is *λ*_D_ = [ε₀ε*k*_B_ *T*/(2*N*_A_ *e* ^2^ *I*)]^½^; substituting ε ≈78.5 and *I* = 150 mol.m⁻^3^ (0.150 M) gives *λ*_D_ ≈0.78 nm (Atkins & de Paula, 2018; Israelachvili, 2011)). As a result, long‐range electrostatic fields are exponentially attenuated in the solvent, and part of the dielectric focusing enhancement is offset by ionic screening.

Notably, the five *Homo sapiens* structures available in the dataset cluster within the negative‐entry group, indicating within‐species consistency among the currently available human structures. The human enzyme remains consistently in the destructive regime upon membrane embedding.

Because ${\Delta \Psi }_{\mathrm{ATP}\;\text{synthase}}$ is approximately zero for *M. tuberculosis* but positive (with larger uncertainty) for *M. smegmatis* in aqueous conditions, the latter may experience a slightly reduced effective electrical driving component under identical membrane potentials. The mycobacterial results may be relevant to ATP‐synthase‐directed drug discovery. Because *M. smegmatis* is frequently used as a surrogate for mycobacterial screening, this difference suggests that responses to oxidative‐phosphorylation inhibitors, including diarylquinolines that target the F_o_/c‐ring region (Matar et al., 2024), may not transfer quantitatively between the two species. The present results do not establish altered drug sensitivity, but they identify a structural‐electrostatic variable that should be considered when interpreting surrogate‐model screens.

Figure 5 provides plots of the axial electrostatic profiles for two representative human ATP synthase structures contributing to Figure 4: 8H9T (rotational state 2, without inhibitor) and 8H9S (rotational state 1, with the endogenous ATPase inhibitor IF1, which prevents ATP hydrolysis under depolarizing conditions (Lai et al., 2023; Shah et al., 2012)). In bulk solvent (left panels), the axial potential difference between the two membrane‐side windows is modest (of the order of 10 mV), consistent with Figure 4 (top). With the low‐dielectric slab (right panels), the potential increases sharply within the slab region, exceeding 200 mV before decaying outside the membrane boundaries. The difference between the same axial windows now yields the amplified values shown for *Homo sapiens* in Figure 4 (bottom). Comparison of 8H9T and 8H9S shows that the inhibitor produces only minor perturbations to the axial electrostatic profile relative to the dominant dielectric amplification introduced by the membrane (Figure 4 (bottom)). This comparison provides an initial assessment of the sensitivity of the human enzyme's axial electrostatic profile to IF1 binding. A systematic ATP/ADP‐state comparison across matched structures remains an important future extension.

**FIGURE 5:**
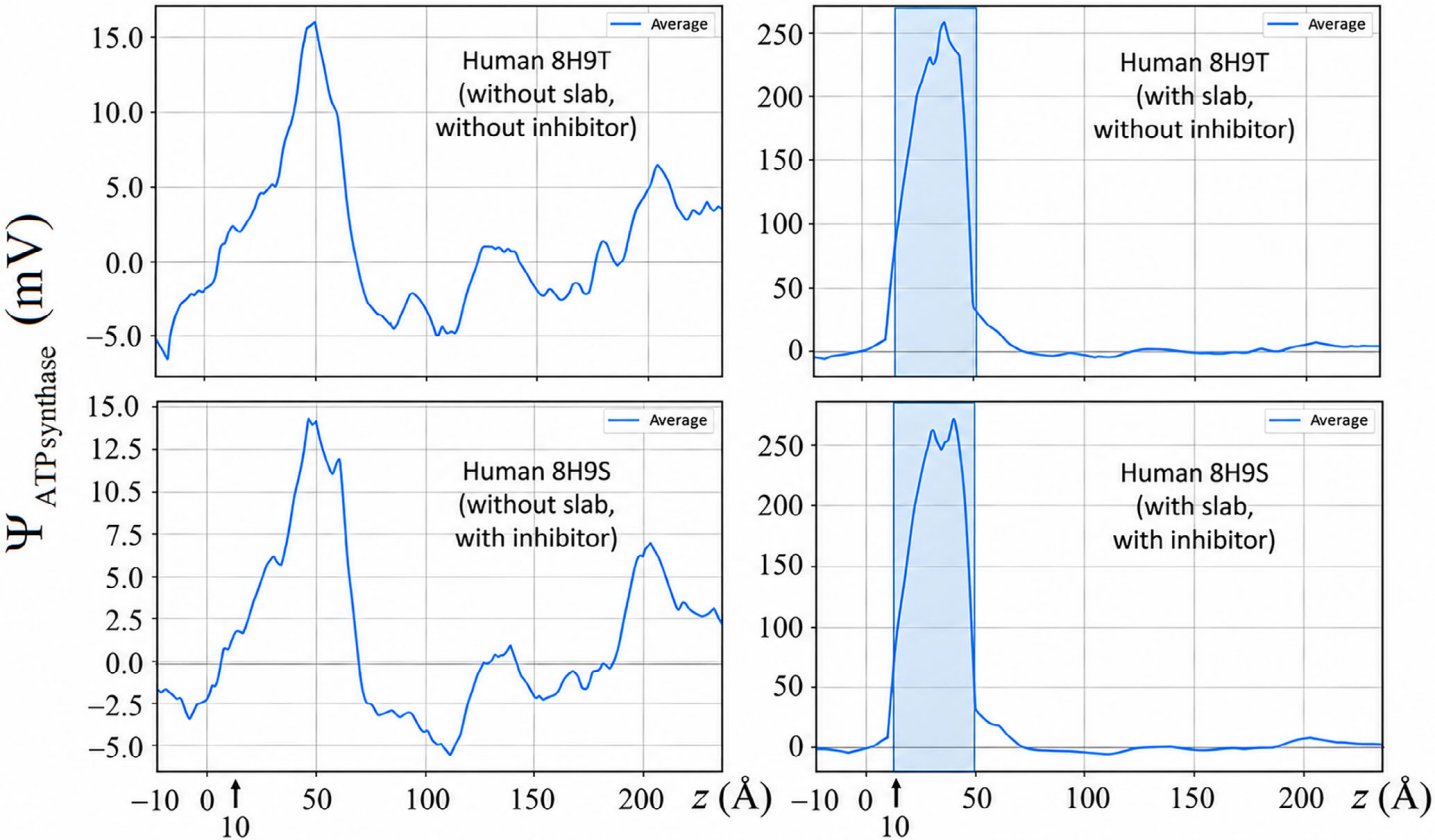
Axial electrostatic potential profiles for representative human ATP synthase structures with and without membrane embedding. Left panels: Plane‐averaged electrostatic potential Ψ(*z*) (mV) along the long molecular axis (*z*) for PDB 8H9T (top, no inhibitor) and 8H9S (bottom, with endogenous inhibitor–see text) in fully aqueous medium (150 mM NaCl, no explicit membrane). Under these conditions, the magnitude of the potential difference between the exit‐side and the entry‐side windows is of the order of 10 mV. Right panels: The same structures after embedding the F_o_ sector in a planar low‐dielectric slab (ε = 4) spanning *z* = +10 to +50 Å (shaded region) to mimic the membrane core. Membrane insertion produces strong dielectric focusing within the slab, with Ψ(*z*) exceeding 250 mV before relaxing outside the membrane region. The enzyme‐linked voltage displayed in Figure 4 is obtained from these profiles as $\Delta \Psi_{\mathrm{ATP}\;\text{synthase}}={\left\langle \Psi \right\rangle }_{\text{matrix}}\;\left(z=+\;50\;Å\;\text{to}\;+70\;Å\right)-{\left\langle \Psi \right\rangle }_{\mathrm{IMS}}\;\left(z=-10\;Å\;\text{to}\;+10\;Å\right)$, see Equation (3), defined analogously to the chemiosmotic membrane potential difference ($\Psi_{\text{matrix}}-\Psi_{\mathrm{IMS}}$). Note the different *y*‐axis scales: Aqueous profiles span approximately ±10 mV, whereas slab‐embedded profiles span ca. >250 mV, an order‐of‐magnitude amplification introduced by the low membrane dielectric constant. The two structures exhibit only minor differences relative to this dominant dielectric effect.

## 3 ENERGETIC SCALE OF THE AXIAL ELECTROSTATIC DESCRIPTOR

The potential differences reported here are static descriptors of the electrostatic landscape generated by the protein charge distribution under defined dielectric and ionic conditions. Nevertheless, the magnitude of the calculated axial potential differences raises a theoretical question: *if part of this protein‐intrinsic electrostatic asymmetry is experienced by a proton during translocation, how large could its energetic effect be?* The following analysis develops a thermodynamic argument for the possible influence of intrinsic protein electrostatics on proton translocation energetics. This hypothesis requires considerable computational and experimental validation. With these qualifications in mind, one may define an effective local electrical potential difference experienced by the proton‐translocation machinery as the sum of the chemiosmotic membrane contribution ($\Delta \Psi_{\text{membrane}}$) and that of the protein itself ($\Delta \Psi_{\mathrm{ATP}\;\text{synthase}}$):

(7) $$\Delta \Psi_{\mathrm{eff}.}=\Delta \Psi_{\text{membrane}}+\;\;\Delta \Psi_{\mathrm{ATP}\;\text{synthase}}.$$

With this new heuristic term, Equation (2) for the Gibbs energy released per mole of protons may be written:

(8) $$\Delta G_{H^{+}}^{\prime }=F\left(\Delta \Psi_{\text{membrane}}+\Delta \Psi_{\mathrm{ATP}\;\text{synthase}}\right)-2.303\;\mathrm{RT}\;\mathrm{\Delta pH}$$

or equivalently:

(9) $$\Delta G_{H^{+}}^{\prime }=F\Delta \Psi_{\mathrm{eff}.}-2.303\;\mathrm{RT}\;\mathrm{\Delta pH},$$

where $\Delta G_{H^{+}}^{\prime }$ is the Gibbs free energy change per mole of translocated protons, including the hypothesized enzyme‐linked electrostatic contribution.

At the reversible threshold for ATP synthesis, *n* protons must collectively supply the Gibbs energy required to synthesize one ATP molecule. Hence, $\Delta G_{H^{+}}^{\prime }=-\Delta G_{\mathrm{ATP}}/n$, where *n* is the number of protons translocated per ATP synthesized. Substituting this relation into Equation (8) yields the required membrane potential to drive ATP formation:

(10) $$\Delta \Psi_{\text{membrane}}=-\frac{\Delta G_{\mathrm{ATP}}}{\mathrm{nF}}+\frac{2.303\;\mathrm{RT}}{F}\;\mathrm{\Delta pH}-\Delta \Psi_{\mathrm{ATP}\;\text{synthase}}.$$

Hence, the sign of $\Delta \Psi_{\mathrm{ATP}\;\text{synthase}}$ determines whether the protein‐intrinsic electrostatic asymmetry would, in principle, reduce or increase the membrane contribution needed to achieve a given ATP‐synthesis free‐energy demand. A contribution with the same enzyme's axial electrostatic orientation as the chemiosmotic electrical term would be constructive, whereas one with the opposite polarity would be destructive. This raises the question whether protein electrostatics could locally bias how the electrical component of the electrochemical gradient is experienced by the protons translocating through the F_o_ motor, without any implication of creation or destruction of free energy by the protein.

To estimate the possible scale of this effect, consider $\Delta G_{\mathrm{ATP}}$ ≈50 kJ·mol^−1^, *n* = 3 H^+^ per ATP, and ΔpH = 0.5. The chemical term contributes 2.303 *RT*ΔpH ≈3 kJ per mole of protons (≈9 kJ per mole of ATP), leaving ~41 kJ·mol^−1^ to be supplied electrically. Dividing by 3*F* yields a required combined electrical potential of ≈0.14 V. An enzyme‐linked contribution of ±20 mV would therefore change the magnitude of the membrane potential required for ATP synthesis by approximately 14% of the calculated 0.14 V electrical requirement. Importantly, *this does not imply the creation or destruction of additional free energy; rather, it redistributes the electrical component between the membrane dielectric and the intrinsic protein field*.

Depending on the metabolic pathway, the maximum P/O ratio (ATP produced per oxygen atom consumed) for an ATP synthase with 10 c‐rings ranges from 1.385 to 2.494 (Brand, 2005). When expressed per molecule of O_2_ rather than per oxygen atom, these values should be doubled. A constructive enzyme‐linked voltage would reduce the membrane contribution required per proton and could, in principle, slightly increase the attainable ATP yield under idealized, tightly coupled conditions. Conversely, a destructive contribution would increase the electrical demand on the membrane potential. In practice, experimentally observed P/O ratios are lower than theoretical maxima due to proton leak and other dissipative processes; thus, the enzyme‐linked term should be interpreted as a modifier of electrical partitioning rather than a determinant of absolute bioenergetic efficiency.

## 4 HUMAN ATP SYNTHASE EXHIBITS AN AXIAL ELECTROSTATIC ASYMMETRY OPPOSING THE MEMBRANE POTENTIAL

Under the convention adopted here ($\Delta \Psi_{\mathrm{ATP}\;\text{synthase}}$ = $\left\langle \Psi_{\text{matrix}}\right\rangle$−$\left\langle \Psi_{\mathrm{IMS}}\right\rangle$), the human enzyme exhibits a positive enzyme‐linked voltage of approximately +0.02 V in the membrane‐embedded model (Figure 4 [bottom]). Because the physiological chemiosmotic membrane potential during ATP synthesis is negative ($\Delta \Psi_{\text{membrane}}$ <0; matrix negative relative to IMS), the human $\Delta \Psi_{\mathrm{ATP}\;\text{synthase}}$ has the opposite sign and therefore partially offsets the membrane potential. In this sense, the enzyme‐linked electrostatic contribution is oriented oppositely to the chemiosmotic driving force. The effective electrical term entering Equations (7)–(9) is therefore:

(11) $$\Delta \Psi_{\mathrm{eff}}\left(\text{human}\right)=\Delta \Psi_{\text{membrane}}+0.02\;V,$$

where $\Delta \Psi_{\text{membrane}}$ <0 under ATP‐synthesis conditions. Thus, under this hypothetical additive model, the magnitude of the net electrical driving voltage would be reduced by ~20 mV relative to the membrane contribution alone.

The corresponding enzyme‐linked contribution to the electrical Gibbs free energy per mole of translocated protons is:

(12) $$\begin{array}{rcl} \Delta G_{\text{elec}} & = & F\;\Delta \Psi_{\text{ATPsynthase}}\\ & \approx & \left(96.5\;\mathrm{kJ}.V^{-1}.\mathrm{mo}l^{-1}\right)\left(0.020\;V\right)\approx 1.9\;\mathrm{kJ}\;\mathrm{mo}l^{-1}. \end{array}$$

For *n* = 3 protons per ATP, this corresponds to ~6 kJ·mol^−1^ less electrical work available for ATP formation; for *n* = 4, the shift is ~8 kJ·mol^−1^.

In this hypothetical model, the enzyme‐linked contribution reflects a redistribution of the electrical component of the proton‐motive force between the membrane potential and intrinsic protein electrostatics. A destructive enzyme‐linked contribution increases the membrane potential magnitude required to reach a given $\Delta G_{\mathrm{ATP}}$. In the illustrative example discussed above (requiring ~0.14 V combined electrical potential), the membrane term must reach ~0.16 V in magnitude if the enzyme contributes +0.02 V.

While the quantitative impact on steady‐state ATP yield depends on coupling efficiency, proton leak, and other dissipative processes (Matar et al., 2025), a 10–20 mV intrinsic offset represents a thermodynamically non‐negligible fraction of the electrical work per proton. Thus, even modest axial electrostatic asymmetries could, if sampled along the F_o_ proton pathway, influence local proton‐transfer energetics.

## 5 POSSIBLE METABOLIC IMPLICATIONS

This section is speculative and is included only to outline possible consequences that could be tested in future work. The static Poisson‐Boltzmann descriptors reported here do not demonstrate altered proton flux, clinical nutrition requirements, body‐mass regulation, or aging‐related mitochondrial decline. These physiological quantities depend on rotary‐state dynamics, protonation equilibria, lipid composition, proton leak, substrate supply, respiratory control, among several other factors. The purpose of this section is to identify where measurable consequences might arise if ATP‐synthase electrostatic asymmetries are later shown to influence local coupling efficiency. The physiological implications discussed below are presented as testable hypotheses rather than established consequences of the present calculations.

The modified Weir equation (with protein correction) expresses the whole‐organism *metabolic rate* (metabolic heat production [MHP]), in watts (Berger et al., 2024; Moonen et al., 2021), commonly inferred from respiratory gas exchange using indirect calorimetry, as (Weir, 1949):

(13) $$\mathrm{MHP}\;\left(W\right)\approx 69.73\;\left(3.94\;{\overset{•}{V}}_{O_{2}}+1.11\;{\overset{•}{V}}_{{\mathrm{CO}}_{2}}-2.17\overset{•}{N}\;\right),$$

where ${\overset{•}{V}}_{O_{2}}$ and ${\overset{•}{V}}_{{\mathrm{CO}}_{2}}$ are the rates of O_2_ consumption and of CO_2_ production (L·min^−1^), and $\overset{•}{N}\;$ is the urinary nitrogen excretion rate (g·min^−1^), accounting for protein oxidation. In Equation (13), the constants 3.94 and 1.11 have units kcal·L^−1^ for O_2_ and CO_2_, respectively; 2.17 has units kcal·g^−1^ for N; and 69.73 W·min·kcal⁻^1^ is the conversion factor from kcal·min^−1^ to watts. Although widely applied in human physiology, this formula is applicable to aerobic organisms with substrate corrections. This relationship reflects substrate oxidation chemistry and respiratory gas exchange. A possible connection to ATP synthase electrostatics would enter only downstream, through the fraction of oxidation energy conserved as ATP versus dissipated as heat, and only if the electrostatic descriptors reported here are shown experimentally to affect coupling efficiency.

The coupling of substrate oxidation to ATP synthesis depends on the proton‐motive force, which comprises both electrical (ΔΨ) and chemical (ΔpH) contributions. If enzyme‐linked ESP modifies the voltage available to the F_o_ motor, the ATP synthesized per molecule of O_2_ reduced may shift slightly under tightly coupled conditions. To represent this possible effect, we may write a phenomenological correction to the classical P/O ratio:

(14) $${\left(\frac{P}{O}\right)}_{\text{effective}}={\left(\frac{P}{O}\right)}_{\text{classical}}\left[\;1+\alpha \;\frac{\Delta \Psi_{\mathrm{ATP}\;\text{synthase}}}{\Delta \Psi_{\text{membrane}}}\;\right],$$

where 0 < α ≤ 1 is a dimensionless empirical coupling/attenuation factor that captures how strongly the enzyme‐linked voltage perturbs the effective voltage drop experienced by the F_o_ motor. For human ATP synthase, ΔΨ_ATP synthase_ > 0, whereas ΔΨ_membrane_ < 0; hence, the correction in Equation (14) is negative, corresponding to a possible reduction in the P/O ratio within this phenomenological model.

For representative mitochondrial values (|ΔΨ_membrane_| ≈150–180 mV), an intrinsic ±20 mV offset constitutes an ≈11–13% potential perturbation of the electrical term before attenuation. The caloric equivalent per liter O_2_ remains unchanged, but the fraction of oxidative energy conserved in ATP versus dissipated as heat may shift slightly if coupling is otherwise tight.

In practice, experimentally observed P/O ratios integrate additional processes such as proton leak, regulatory modulation, and structural variability (Matar et al., 2025). The enzyme‐linked voltage identified here should therefore be regarded as one contributor to energetic partitioning rather than a primary determinant of organismal metabolic rate. Animal physiology often notes that some species or conditions yield slightly different caloric equivalences. For instance, trained athletes can exhibit improved efficiency, burning slightly less O_2_ for the same mechanical work output. While some of that is due to biomechanical efficiency (Jacobs & Lundby, 2013; Layec et al., 2016), it does not exclude that possibly a part of it could be traced to mitochondrial coupling efficiency. These findings point to the possibility that species differences in ATP synthase's electrostatic potential might underlie some variations in basal metabolic rate or thermogenesis.

Clinical nutrition commonly expresses substrate energy content using empirical caloric factors (e.g., 4 kcal·g^−1^ for carbohydrate, 9 kcal·g^−1^ for fat–the so‐called Atwater factors, which are not directly applicable to total parenteral nutrition (TPN)), reflecting the metabolizable enthalpy of oxidation after digestive and urinary corrections (Tontisirin, 2003). The ATP yield from substrate oxidation also depends on mitochondrial coupling, as illustrated by the following energy balance:

(15) $$-\Delta G_{\text{substrate oxidation}}\approx \left(\mathrm{ATP}\;\text{yield}\right)\times \Delta G_{\mathrm{ATP}}+\text{T}\text{△}{\text{S}}_{\text{gen}},$$

assuming isothermal conditions and neglecting other forms of energy storage or useful work, and where Δ*G*_substrate oxidation_ < 0 since substrate oxidation is exergonic, Δ*G*_ATP_ > 0 since the synthesis of ATP consumes free energy, and *T*Δ*S*_gen_ is the free energy irreversibly dissipated through the generation of entropy. If the effective proton free energy is reduced by an opposing $\Delta \Psi_{\mathrm{ATP}\;\text{synthase}}$ >0 then the ATP/O_2_ ratio may be reduced. This could result in fewer ATP molecules being generated per mole of substrate oxidized, even though the substrate's metabolizable energy content (kcal/g) remains, of course, unchanged. In practical terms, caloric adequacy and ATP‐generating capacity need not be identical in individuals with reduced mitochondrial coupling efficiency. The present study does not show that ATP‐synthase electrostatics measurably affects TPN response. Instead, it identifies *one molecular variable* that could be examined in future studies of coupling efficiency.

While the estimated perturbation of the electrical component is modest (on the order of ~10%), in critically ill patients, neonates, cachectic patients, or those with mitochondrial disorders, the reduction in ATP yield per kcal could become clinically significant (Brealey et al., 2002; DiMauro & Schon, 2003; Tisdale, 2009). Whether this contributes to clinical observations such as failure to thrive despite apparent caloric sufficiency remains an open question requiring direct clinical and bioenergetic testing.

Could intrinsic electrostatic modulation of ATP synthase alter the partitioning of dietary energy between ATP synthesis and heat, thereby influencing the relationship between caloric intake and net energy storage? This would occur if the same caloric intake resulted in a greater fraction of oxidation energy being dissipated as heat rather than conserved in ATP, holding behavior, appetite, and activity fixed. In this case, the net energy expenditure could be higher; hence, in humans, for example, weight gain per labeled “(food) Calorie”, 1 Calorie = 1000 calorie, could be lower.

The enzyme‐linked offset is on the order of 10–20 mV, while the mitochondrial membrane potential magnitude is typically ~150‐180 mV, thus under the hypothetical additive model, the electrical driving term relevant to oxidative phosphorylation could be perturbed at the level of roughly 6–13% before attenuation. However, the effect on whole‐body energy balance need not be proportional to this perturbation because coupling is already influenced by proton leak, substrate cycling, variable P/O, and regulated thermogenesis. Hence, the effective impact on total daily energy expenditure could be much smaller.

We introduce an organism‐level phenomenological attenuation factor *η*, 0 < *η* ≤ 1, that quantifies how much of a mitochondrial‐scale perturbation in energy partitioning (here, an intrinsic $\Delta \Psi_{\mathrm{ATP}\;\text{synthase}}$ offset) is expressed in net whole‐body energy balance after behavioral and physiological compensation. No direct empirical estimate of *η* exists for this specific electrostatic mechanism; we therefore examine an illustrative range informed by two physiological considerations. First, exercise‐intervention studies demonstrate substantial variability in physiological energy compensation, which may increase with intervention duration(Riou et al., 2015). These observations illustrate the potential importance of organism‐level compensation, without directly constraining *η*. Second, mitochondrial proton leak can account for approximately 20–30% of resting respiration in certain tissues (Brand, 1990), although this does not establish an upper bound on *η*. For illustration, we adopt *η*≈0.05–0.2.

As an illustrative calculation, the product of a mitochondrial‐scale electrical perturbation (0.06–0.13) and an organism‐level attenuation factor *η* (0.05–0.2) would yield approximately 0.3–2.6% of daily energy flux. For an intake of 2000 kcal·day^−1^, this corresponds to roughly 6–52 kcal·day^−1^. Using ~7700 kcal as the energy equivalent of 1 kg of adipose tissue (Thomas et al., 2014), a persistent and uncompensated difference of 6 kcal·day^−1^ would amount to ~1 kg over approximately 1280 days (~3.5 years), whereas 52 kcal·day^−1^ would correspond to ~1 kg over ~150 days (~5 months). In practice, appetite regulation, spontaneous activity, endocrine responses, and adaptive thermogenesis would be expected to attenuate much of this theoretical imbalance, so any influence of intrinsic ATP synthase electrostatics would most plausibly manifest as a subtle shift in long‐term energy partitioning rather than a major determinant of body mass trajectory.

Over long time scales, small differences in ATP/O_2_ yield could, in principle, interact with age‐related changes in mitochondrial efficiency, but the present calculations do not establish such a mechanism. Mitochondrial coupling and oxidative phosphorylation capacity are known to decline with aging and in several chronic diseases, including metabolic syndrome and neurodegeneration, often accompanied by increased proton leak and altered respiratory control (Bratic & Larsson, 2013; Harman, 1972; Petersen et al., 2003; Sun et al., 2016). Because the intrinsic electrostatic profile of ATP synthase arises from the spatial distribution of charged residues and local dielectric environment, it is unlikely to be identical across tissues, physiological states, or individuals. Subtle, persistent differences in intrinsic $\Delta \Psi_{\mathrm{ATP}\;\text{synthase}}$, whether due to genetic variation, somatic mutation, oxidative damage, or altered membrane composition, could therefore contribute to small but cumulative shifts in ATP production efficiency and thermogenic balance over the human lifespan. We emphasize that this remains a testable hypothesis rather than a demonstrated mechanism; direct experimental quantification of ATP synthase electrostatic variation in aging and disease states will be required.

## 6 LIMITATIONS

Several limitations should be emphasized. First, the electrostatic potentials reported here are static continuum quantities obtained from Poisson–Boltzmann calculations on fixed protein structures; they do not describe explicit proton hopping, hydration dynamics, conformational fluctuations, or the time‐dependent membrane potential. It is also worth emphasizing that the present calculations do not establish rotary‐state dependence. The c‐ring has approximate *C_n_* pseudosymmetry (e.g., *n* = 8 in *Homo sapiens*), whereas the α_3_β_3_ catalytic head has approximate *C*_3_ pseudosymmetry and the γ central stalk is asymmetric. This symmetry mismatch can result in phase‐dependent electrostatic variations accompanying the rotation near the proton half‐channels. The present axial profiles should therefore be read as static structural descriptors and not as time‐averaged electrostatic fields over a full catalytic cycle.

Second, the plane‐averaged potentials are global axial descriptors and should not be equated with the free‐energy profile along the actual F_o_ proton‐translocation pathway, which involves localized half‐channels, protonatable residues, and transient hydrogen‐bonded networks. Third, protonation states assigned by PROPKA at pH 7.0 are approximate and may differ under membrane potential, lipid‐specific, or conformationally distinct conditions. Fourth, the membrane model used here is a simplified low‐dielectric slab and does not include explicit lipid headgroups, cardiolipin enrichment, surface charge, membrane dipoles, or bilayer asymmetry. Thus, the reported ΔΨ values should be interpreted as comparative, order‐of‐magnitude structure‐derived electrostatic descriptors rather than independently measurable membrane voltages or demonstrated additive components of the proton‐motive force. Their functional relevance will require pathway‐resolved electrostatic sampling, molecular dynamics, constant‐pH calculations, mutagenesis, and direct biochemical or bioenergetic validation. Considerable further work is therefore needed before the proposed functional role of these electrostatic asymmetries can be firmly established.

## 7 CONCLUSIONS

We provide a comparative electrostatic analysis of 178 experimentally determined ATP synthase structures from 17 species. Across this dataset, ATP synthase exhibits reproducible species‐dependent axial electrostatic asymmetries, with potential differences of the order of 10–20 mV between defined membrane‐proximal axial windows in several taxa. These differences arise from the spatial distribution of charged residues, dielectric boundaries, and structural organization of ATP synthase.

The observed profiles reveal substantial electrostatic diversity among ATP synthases. In particular, the five available *Homo sapiens* structures show a consistent axial electrostatic orientation within the human dataset. Broader variation is observed when comparing ATP synthases across the full set of 17 species. These observations reflect two distinct levels of comparison: within‐species structural consistency for *Homo sapiens* and across‐species electrostatic diversity for the broader dataset. The mycobacterial comparison is also noteworthy: *M. tuberculosis* shows a near‐zero axial electrostatic asymmetry, whereas *M. smegmatis* exhibits a more positive axial electrostatic asymmetry and is more variable. These differences may be relevant to future studies of proton‐pathway energetics, species‐specific coupling, and ATP‐synthase inhibitors.

The possible functional significance of these electrostatic asymmetries remains a hypothesis to be confirmed by future investigations. If such protein‐intrinsic potential differences persist near the F_o_ proton half‐channels during the catalytic cycle, they could influence local protonation, proton release, proton transfer, or inhibitor binding. They should not, on the basis of the present static calculations alone, be treated as demonstrated additive components of the pmf. Testing this hypothesis will require pathway‐resolved electrostatic calculations, molecular dynamics, constant‐pH simulations, targeted mutagenesis of residues in the a‐subunit and c‐ring region, and biochemical measurements of ATP synthesis, proton flux, membrane potential, or inhibitor response.

More broadly, the present analysis shows that comparative protein electrostatics can reveal conserved and divergent features of large rotary enzymes that are not immediately apparent from structural coordinates alone. These results provide an electrostatic atlas of ATP synthase and a set of experimentally and computationally testable hypotheses concerning how protein charge architecture may shape local energy‐transduction landscapes.

## 8 METHODS

### 8.1 Dataset collection

A total of 178 unique F_o_‐F_1_‐ATP synthase structures were retrieved from the Protein Data Bank (PDB), representing 17 distinct species. This set encompasses the publicly available high‐resolution structures of F‐type ATP synthase at the time of data collection. Only experimentally solved structures were considered; predicted models (e.g., AlphaFold entries) were excluded. Structures were retained when the experimentally determined model was sufficiently complete to define the F_o_F_1_ complex, the c‐ring/rotor axis, and the axial averaging windows reproducibly (entrance/IMS: z=−10 to +10 Å, exit/matrix: z=+50 to +70 Å). No single hard resolution cutoff was imposed. Because continuum electrostatic calculations can be sensitive to local structural details, the reported values should be interpreted as comparative descriptors obtained under a uniform preparation protocol.

### 8.2 Workflow overview

All computational analyses were performed using a Nextflow‐based workflow (*ATPase_EP_Analyzer.nf*) developed in‐house to ensure reproducibility and scalability on high‐performance computing resources (Matar & Matta, 2025a). The workflow orchestrates multiple Python modules and external tools, managing structure preprocessing, coordinate alignment, electrostatic potential calculations, and data aggregation.

Unlike the dipole moments of charged proteins, which depend on the choice of coordinate origin (Matar & Matta, 2025b), electrostatic potential differences are origin‐independent. Nevertheless, our cross‐species comparisons of ΔΨ_ATP synthase_ were performed using a consistently defined membrane‐normal axis and identical axial sampling windows (Matar & Matta, 2025c).

### 8.3 Reproducibility

To facilitate reproducibility and to make the electrostatic protocol fully transparent, the main computational parameters used throughout the study are summarized in Table 1. These include the structural dataset, preparation workflow, protonation protocol, force‐field assignment, dielectric constants, ionic conditions, membrane‐slab model, coordinate‐alignment convention, axial averaging windows, and software environment. The purpose of the table is to make explicit that all 178 ATP synthase structures were treated under the same electrostatic, geometric, and numerical assumptions, so that the reported differences reflect comparative structure‐dependent trends rather than changes in computational setup.

**TABLE 1:** Computational parameters used for electrostatic‐potential calculations.

Parameter	Value or procedure used
Structural database	RCSB Protein Data Bank
Number of ATP synthase structures	178
Number of species	17
Structure type	Experimentally determined crystallographic and cryo‐EM structures
Computationally‐predicted structures	Excluded (only experimental structures are included)
Structure preparation	Missing residues and/or heavy atoms completed where required before electrostatic analysis
Hydrogen atoms	Added during PDB2PQR/PROPKA preparation
Protonation‐state assignment	PROPKA at pH 7.0
Force‐field parameters	AMBER charges and atomic radii assigned by PDB2PQR
Electrostatic solver	Adaptive Poisson–Boltzmann Solver (APBS)
Poisson–Boltzmann model	Linearized Poisson–Boltzmann equation
Temperature	298 K
Solvent dielectric constant	ε_solvent_ = 78.5
Protein dielectric constant	ε_protein_ = 6.0
Ionic strength	150 mM NaCl
Sodium‐ion radius	Na^+^ = 2.0 Å
Chloride‐ion radius	Cl^−^ = 1.8 Å
Membrane model, when used	Planar low‐dielectric slab
Membrane dielectric constant	ε_membrane_ = 4.0
Membrane slab position	*z* = +10 to +50 Å
Coordinate alignment	Structures aligned to a common protein‐fixed coordinate frame using the c‐ring/rotor axis as the *z*‐axis reference
Main axial descriptor	Plane‐averaged electrostatic potential, ⟨Ψ(*x*,*y*|*z*)⟩
Entry‐side averaging window	*z* = −10 to +10 Å
Exit‐side averaging window	*z* = +50 to +70 Å
Reported electrostatic difference	ΔΨ = ⟨Ψ⟩_exit‐side_ − ⟨Ψ⟩_entry‐side_
Analysis region	Solvent‐accessible regions within the selected axial slices; protein‐proximal masking applied as described in Methods
Workflow	In‐house Nextflow/Python pipeline
Main software environment	Python 3.12, PyMOL‐open‐source, PDBFixer, APBS, SciPy, mmCIF‐PDBx, OpenMM, matplotlib, NumPy
Reproducibility	Workflow modules log inputs, outputs, and errors; code and analysis scripts are made available through the project repository

The complete pipeline was executed under a Conda environment (Python 3.12, PyMOL‐open‐source, PDBFixer, APBS, SciPy, mmCIF‐PDBx, OpenMM, matplotlib, NumPy). All steps were automated under Nextflow, enabling parallelization across multiple structures. Each module logs outputs and errors to ensure full reproducibility.

### 8.4 Structural preprocessing and alignment

1. Rotor (the c‐ring) isolation and alignment were performed using a PyMOL‐based script (*pymol_c_ring_aligner.py*), which automatically:

- Identifies the c‐ring helices in each structure via MMCIF parsing,
- constructs pseudoatoms to define Cartesian reference axes,
- generates centroids (*centroid.py*) to approximate the c‐ring central axis,
- aligns the rotor relative to the stator using pseudoatom vectors,
- outputs aligned whole ATP synthase and isolated c‐ring PDB files for subsequent steps.

2 Protonation state assignment and electrostatic input generation were carried out with a wrapper script around PDB2PQR (*pdb2pqr.py*):

- Force field: AMBER.
- Protonation: PROPKA at physiological pH (7.0).
- Ion parameters: 150 mM NaCl (Na^+^ radius 2.0 Å, Cl^−^ radius 1.8 Å).
- Water dielectric constant: ε = 78.5.
- Protein dielectric constant: ε = 6.0.

3 APBS input files were automatically modified to include ion settings and standardized potential output (*‐pot.dx). For calculations with the membrane, a dielectric slab representing the biological membrane was added.

### 8.5 Electrostatic potential calculation and analysis

The resulting ESP maps were processed with the analysis module (*apbs_ctscan_analysis.py*), which implements the following procedure:

- Computation of the three‐dimensional electrostatic potential Ψ_ATP synthase_(*x*,*y*,*z*) maps for each ATP synthase structure using APBS (*Adaptive Poisson–Boltzmann Solver*). APBS was executed on the PQR file with its custom “.*in*” parameters file generated by *pdb2pqr.py*.
- For calculations with the membrane, the generated maps are modified to introduce a 40 Å membrane slab (ε = 4.0) dielectric model using *draw_membrane2a*, then the modified maps are resubmitted to APBS to perform the complete system simulation. The slab thickness is centered on the F_o_ rotor's *z*‐axis, spanning the rotor‐stator interface from *z* = 10 Å to 50 Å.
- Protein geometric analysis is performed (*prot_dim*) to locate the geometric midpoint of the F_o_ rotor.
- Slicing of the ESP grid (*ct_scan*) as a series of planar cross‐sections perpendicular to the enzyme's long axis (the *z*‐axis). Each section represents a two‐dimensional map of Ψ_ATP synthase_(*x*,*y*) at a fixed *z*‐coordinate, allowing direct inspection of the potential distribution in planes perpendicular to the *z*‐axis.
- A masking function (*mask_pot*) is implemented to restrict the analysis to solvent regions between 5 Å and 15 Å from the protein surface.
- The *z*‐component of the electric field is estimated as the negative numerical derivative of the electrostatic potential with respect to *z* (*E_z_* = −∂Ψ/∂*z*), (*efield*).
- Angular averaging is applied (*ang_avg*) over circular slices to yield the mean ESP, standard deviations, and extrema as a function of *z*. Sectorized angular averages are also recorded.

The output of the procedure described above includes:

- Slice‐averaged ESP curves (mean ± standard deviation).
- A set of planar sections of the Ψ_ATP synthase_(*x*,*y*) perpendicular to the long protein axis (*z*‐axis) expressed numerically (two‐dimensional grids) and visualized graphically for visual inspection.
- Summary data files for each structure containing *z*‐averages (with their in‐plane standard deviations)

From the potential slices, potential differences (voltages) between the out/proton entry side and the in/proton exit side regions are then estimated.

## AUTHOR CONTRIBUTIONS

**Islam K. Matar:** Conceptualization; investigation; writing – original draft; methodology; validation; visualization; writing – review and editing; software; formal analysis; data curation. **Peyman Fahimi:** Investigation; writing – original draft; validation; writing – review and editing; formal analysis. **Jean‐Nicolas Vigneau:** Investigation; methodology; software. **Chérif F. Matta:** Conceptualization; investigation; funding acquisition; writing – original draft; methodology; validation; visualization; writing – review and editing; formal analysis; project administration; supervision; resources; software.

## DECLARATION OF GENERATIVE AI AND AI‐ASSISTED TECHNOLOGIES IN THE MANUSCRIPT PREPARATION PROCESS

During the preparation of this work the authors used ChatGPT in order to condense and improve the writing structure in some passages. After using this tool/service, the authors reviewed and edited the content as needed and take full responsibility for the content of the published article.

## Supporting information


**DATA S1.** Python analysis notebook exported as PDF, showing the post‐processing workflow for APBS‐derived electrostatic‐potential CSV files, including data loading, exclusion of non‐F_o_F_1_ ATP synthase or otherwise unsuitable entries, classification of ATP synthase structures by axial electrostatic‐potential profiles at the proton entry window, and generation of the combined and individual profile plots supporting the comparative electrostatic analysis.

## Data Availability

The experimentally determined ATP synthase structures analyzed in this study are publicly available through the RCSB Protein Data Bank (https://www.rcsb.org/). The computational workflow is available at https://github.com/Orbitaliant/ATPase‐EP‐Analyzer, and the Supporting Information provides analysis code and individual electrostatic‐potential profiles.
